# Aromatase deficiency due to novel *CYP19A1* mutation: a rare cause of maternal and fetal virilization

**DOI:** 10.1515/crpm-2023-0032

**Published:** 2024-04-26

**Authors:** Aamir Naseem, Muhammad Zahid, Kashan Arshad, Syed Saddam Hussain, Sommayya Aftab, Anjum Saeed, Huma Arshad Cheema

**Affiliations:** Department of Pediatric Endocrinology and Diabetes, 66867 University of Child Health Sciences, The Children’s Hospital Lahore, Lahore, Pakistan; Department of Pediatric Gastroenterology, Hepatology and Nutrition, 66867 University of Child Health Sciences, The Children’s Hospital Lahore, Lahore, Pakistan

**Keywords:** aromatase deficiency, atypical genitalia, maternal virilization, novel mutation, *CYP19A1*

## Abstract

**Objectives:**

Aromatase deficiency is a rare autosomal recessive condition due to a mutation in the *CYP19A1* encoding aromatase enzyme. This enzyme protects the fetus and mother from excess androgens by converting them into estrogen. We are reporting a case of aromatase deficiency presented with atypical genitalia and maternal virilization due to a novel mutation in *CYP19A1*.

**Case presentation:**

A 10-day-old newborn presented with atypical genitalia and a history of maternal virilization during pregnancy. On examination, the baby had a Prader score of 3. Further investigation revealed karyotype 46 XX, with a normal uterus and ovaries on ultrasonography. The hormonal profile of the baby was normal except for the raised follicle stimulating hormone (FSH). Maternal ultrasound revealed polycystic ovaries, and the hormonal profile was within the normal range with slightly raised testosterone. Whole exome sequencing was done, which reported that the baby was carrying a novel homozygous mutation of the *CYP19A1* gene c.575G>C p. (Arg192Pro), confirming the diagnosis of aromatase deficiency.

**Conclusions:**

Aromatase deficiency is a rare condition. A history of maternal virilization during pregnancy in a child born with atypical genitalia should alert physicians to consider aromatase deficiency.

## Introduction

The aromatase enzyme is a member of the cytochrome p450 superfamily, which is encoded by *CYP19A1 located* on the 15q21.1 chromosome. It is a crucial enzyme for estrogen biosynthesis and is expressed abundantly in many tissues, such as testes, ovaries, adipose tissue, placenta, brain, and skin. In gonadal tissue, aromatase converts androstenedione and testosterone into estrone and estradiol, respectively, which are responsible for breast development in females and epiphyseal closure in males. In placental tissue, it converts 16-hydroxy dehydroepiandrosterone sulphate into estriol to prevent the accumulation of androgens in mother and baby. So, a lack of aromatase activity during pregnancy results in an excess of 16-hydroxy dehydroepiandrosterone, which is further converted into androgens by the three beta-hydroxysteroid dehydrogenase enzymes. This excess of androgens leads to maternal and fetal virilization (female atypical genitalia) [1, 2].

Aromatase deficiency is an autosomal recessive condition due to mutations in the *CYP19A1* gene. More than 30 mutations have been reported so far. It usually presents with signs and symptoms of maternal virilization, such as cystic acne, clitoromegaly, hirsutism, and voice deepening during pregnancy. It also results in the virilization of the external genitalia of the female fetus [1, 2].

We are reporting a rare case report of a neonate with aromatase deficiency due to a novel mutation in *CYP19A1*, c.575G>C p. (Arg192Pro) from Pakistan.

## Case presentation

**Figure 1:**
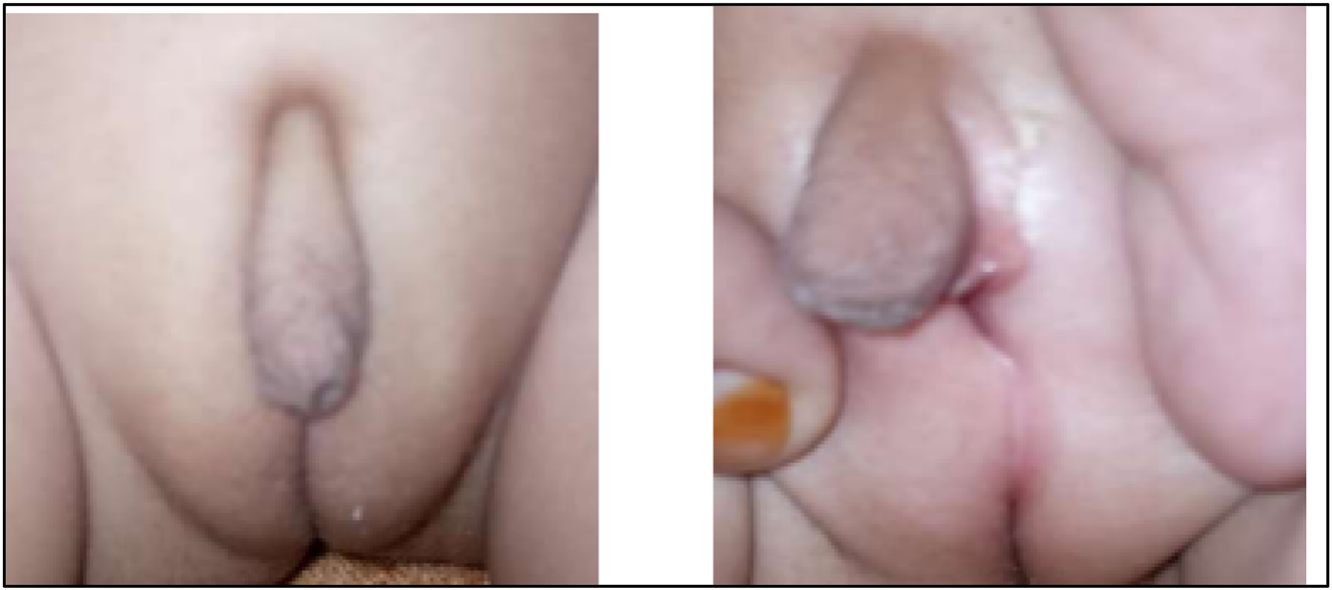
Showing atypical genitalia of baby.

A 10-day-old neonate presented to the department of pediatric endocrinology and diabetes, University of Child Health Sciences, The Children’s Hospital, Lahore, with concern for atypical genitalia. The baby was born full term through spontaneous vaginal delivery with a birth weight of 3.8 kg. The parents were consanguineous with one older male healthy sibling. The baby was thriving well with no concern for loose motion, vomiting, dehydration, or increased pigmentation. There was no family history of atypical genitalia, neonatal or infant death, or congenital adrenal hyperplasia. On probing, there was a significant history of maternal virilization during pregnancy in the form of severe acne, hirsutism, and hoarseness of voice (Figure 1).

**Figure 2:**
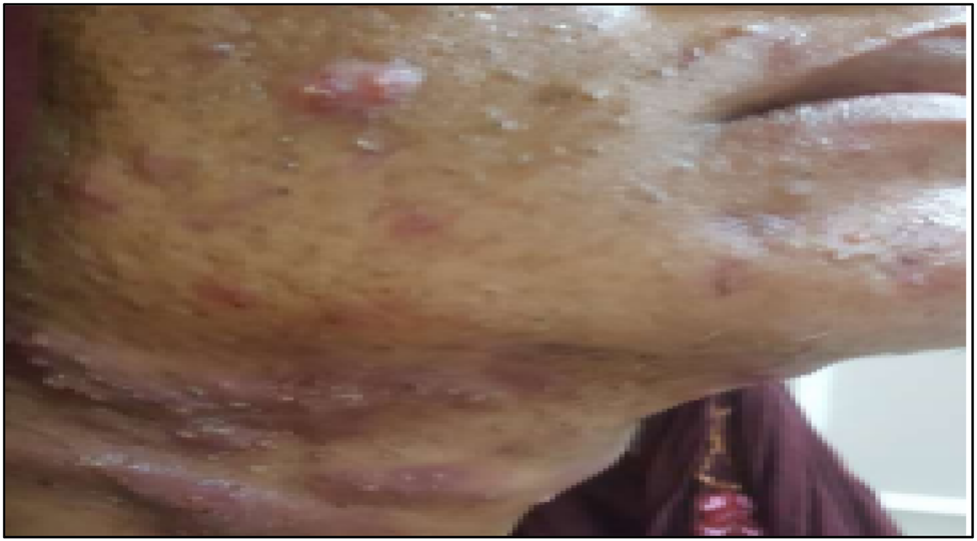
Showing severe pustular acne on face of mother.

On examination, the baby was well-thriving with no signs of dehydration or pigmentation. Blood pressure in both the baby and mother was normal. Genital examination revealed a phallus length of 2 cm, posteriorly fused labio-scrotal folds, a single urogenital opening, and impalpable gonads, as shown in Figure 2.

Detail investigations showed karyotyping 46 XX, with ultrasound pelvis showing bilateral ovaries, uterus, and fallopian tubes. No evidence of testis was found. The hormonal profile of the baby revealed normal sodium, potassium, 17-hydroxy progesterone, and cortisol. Baby luteinizing hormone (LH) was normal for age (5.5 IU/L); however, follicle stimulating hormone (FSH) was markedly raised (44.21 IU/L) with undetectable estradiol, anti-Müllerian hormone (AMH), and testosterone, as shown in Table 1.

**Table 1:** Hormonal profile of the baby.

Investigations	Result
Electrolytes	Normal
17- OH progesterone	4.87 ng/mL
Cortisol	21.18 μg/dL
LH	5.5 mIU/mL
FSH	44.21 mIU/mL
Estradiol	Undetectable
DHEA sulphate	52.0 μmol/L
Testosterone	<2.50 μg/dL
AMH	0.005 ng/mL

LH, luteinizing hormone; FSH, follicle stimulating hormone; DHEA, dehydroepiandrosterone; AMH, anti-Müllerian hormone.

Maternal workup showed no evidence of adrenal or ovarian tumors on ultrasonography; however, there were bilateral multiple ovarian pericapsular follicles, which seemed to mimic bilateral polycystic ovaries. The hormonal profile of the mother was normal, as shown in Table 2, with no evidence of maternal congenital adrenal hyperplasia (CAH).

**Table 2:** Hormonal profile of the mother.

Investigations	Result
Electrolytes	Normal
ACTH	17.4 pg/mL
Cortisol	26.8 μg/dL
17- OH progesterone	0.17 ng/mL
DHEA sulphate	35.70 g/dL
Testosterone	6.65 μg/dL

ACTH, adrenocorticotropic hormone; DHEA, dehydroepiandrosterone.

Baby whole exome sequencing reported that the baby is carrying a novel homozygous variant of the *CYP19A1* gene c.575G>C p. (Arg192Pro), with both parents being carriers, confirming the diagnosis of autosomal recessive aromatase deficiency.

The family was counselled about the condition and reassured that the baby does not need any medical treatment now but needs regular monitoring. The baby had a cystoscopy showing a 1.5 cm common channel, and a plan was made for feminizing genioplasty. Maternal acne improved with time. Currently, the baby is two years old and thriving well with LH 1.02 IU/L, FSH 67.9 IU/L, and testosterone <2.5 ng/dL, with bilateral ovaries showing tiny follicles. In the last follow-up visit, oral conjugated estrogen at 0.15 mg/day was started.

## Discussion

Aromatase deficiency is a very rare condition caused by the *CYP19A1* mutation, leading to the impaired conversion of androgens into estrogens. It was first defined in 1991 by Shozu et al., and till date, few cases have been reported with more than 30 mutations in *CYP19A1*. Most of these mutations are in exons 10 and 9 [1].

The human *CYP19A1* (p450arom) is in the 21.2 region on the long arm of chromosome 15 (15q21.2). This gene spans a region that consists of a 30 kb coding region and a 93 kb regulatory region (approximately 123 kb total length). Its regulatory region contains at least 10 distinct promoters regulated in a tissue or signaling pathway-specific manner [2]. In humans, cP450 aromatase is the product of the gene. The protein-coding sequence is contained within nine exons (2–10) that span approximately 35 kb of DNA [3].

Aromatase deficiency in the placenta blocks the conversion of androgens to estrogen, leading to androgen accumulation in both the baby and the mother. This increased concentration of androgens during fetal life results in varying degrees of virilization of the external genitalia of the female baby. The external genitalia of male babies remain unaffected. During pre-pubertal life, males remain asymptomatic, but females can have multiple cysts in the ovaries due to a lack of inhibition of gonadotropins, especially FSH, by estrogen. As puberty kicks in and the hypothalamo-pituitary-gonadal axis gets activated, a lack of aromatase enzyme activity leads to signs and symptoms of accumulation of androgens (virilization) and a lack of estrogen (no thelarche, primary amenorrhea, and hypergonadotropic hypogonadism). The estrogen deficiency also results in delayed epiphyseal closure, osteopenia, eunuchoid body proportion, and osteoporosis in both genders [4].

In most fetuses with aromatase deficiency, maternal virilization can be seen as early as 12 weeks or as late as up to 30 weeks of gestation. Signs of maternal virilization such as acne, hirsutism, clitoromegaly, and hoarseness of voice gradually disappear postnatally as the androgen levels return to the normal [5]. Clinical findings in aromatase deficiency vary depending on the retained proportion of enzyme function. Placental aromatase activity of as little as 1–2 % is reported to be protective against maternal virilization during pregnancy [6]. In aromatase-deficient girls, basal and GnRH-stimulated FSH levels were far greater than in normal subjects, whereas estradiol and estrone were either too near the detection limit of radioimmunoassay or undetectable [7].

Medical management of aromatase deficiency in females includes estrogen replacement therapy, which can be initiated as early as two years of age if there is evidence of polycystic ovaries suppressing gonadotropins. This treatment should be initiated and sustained with the lowest possible dose of estrogen possible to prevent the development of ovarian cysts and to avoid the early development of breasts and the acceleration of bone age. It is recommended to start with oral conjugated estrogens (0.15 mg/day or every other day) or micronized estradiol (0.25 mg/day or every other day) with the aim of maintaining the suppression of FSH and LH [8].

At the time of puberty, girls should increase estradiol to 0.3 mg/day with a gradual increment to achieve breast development. Once there is breakthrough bleeding or two years of estrogen therapy, add progesterone. Boys also need low-dose estrogen at the time of puberty for epiphyseal closure and prevention of metabolic derangement due to a lack of estrogen [7].

## Conclusions

Aromatase deficiency is a very rare condition. We are reporting a case of aromatase deficiency due to a novel mutation. A history of maternal virilization in babies born with atypical genitalia should alert the physician to consider aromatase deficiency.
